# Supplementary material for: Neurophysiological Effects of Withdrawal from Acute Overused Medications in Chronic Migraine with Medication-Overuse Headache
Source: J Clin Med. 2024 Dec 9;13(23):7491. doi: 10.3390/jcm13237491 (PMC11642649; doi:10.3390/jcm13237491)
Supplement: Supplementary file 1 [file jcm-13-07491-s001.zip › jcm-3306665-supplementary.pdf]

Table S1. Correlation between significant electrophysiological data at T_1 and clinical variables at T_1

		Hab(1-2) _T₁	pre-HFO amp_T₁	Post-HFO amp_T₁
**Att Freq_T_1**	Pearson's r	-0.72	-0.00	0.25
	df	12	12	12
	p-value	0.004	0.993	0.391
**Tablets_T_1**	Pearson's r	0.13	-0.12	0.13
	df	12	12	12
	p-value	0.667	0.683	0.651
**Abuse phase (years)**	Pearson's r	0.14	-0.49	0.06
	df	12	12	12
	p-value	0.632	0.073	0.839
**Chronic phase (years)**	Pearson's r	0.19	-0.48	0.16
	df	12	12	12
	p-value	0.518	0.082	0.588
**Duration_ T_1**	Pearson's r	0.26	-0.20	-0.31
	df	12	12	12
	p-value	0.374	0.500	0.279
**VAS_T_1**	Pearson's r	0.28	-0.10	-0.36
	df	12	12	12
	p-value	0.325	0.737	0.208
